# Causes and 3-year-incidence of blindness in Jing-An District, Shanghai, China 2001-2009

**DOI:** 10.1186/1471-2415-11-10

**Published:** 2011-05-05

**Authors:** Liangcheng Wu, Xinghuai Sun, Xingtao Zhou, Chenghai Weng

**Affiliations:** 1 Jing-An District Central Hospital, Shanghai, China; 2 Eye and ENT hospital, Shanghai Medical College, Fundan University, Shanghai, China

## Abstract

**Background:**

Registered data can provide valuable information regarding blindness. The purpose of this study was to evaluate the main causes and 3-year incidence of registered blindness in Jing-An district in Shanghai, China.

**Methods:**

Data from the blindness registry (age, gender and cause of visual disability) were collected and analyzed. The prevalence of blindness for 2003, 2007, 2009 and the 3-year incidence of blindness were calculated.

**Results:**

The reported blindness increased significantly from 113.7 per 100,000 in 2003 to 145.8 per 100,000 in 2006 to 165.9 per 100,000 in 2009 (P < 0.05, P < 0.05, respectively). Age significantly affects prevalence; the odd ratios (OR) were 2.57 in the 30 y - 49 y range (P < 0.001), 7.27 in the 50 y - 69 y range (P < 0.001) and 21.2 in the ≥ 70 y (P < 0.001). The 3-year incidence increased from 32.3 per 100,000 in 2001-2003 to 34.2 per 100,000 in 2004-2006 to 40.8 per 100,000 in 2007-2009. The causes of new blindness registered in 2001-2009 were myopic macular degeneration (19.4%), followed by glaucoma (17.7%), age-related macular degeneration (11.8%), optical nerve atrophy (9.4%), retinitis pigmentosa (8.6%), diabetic retinopathy (7.8%) and corneal opacity (5.8%).

**Conclusions:**

The 3-year incidence and prevalence of registered blindness increased in the past 9 years. The leading causes of new blindness were myopic macular degeneration, glaucoma and age-related macular degeneration. The pattern of causes has changed little in the past 9 years and is different from other locations in China. The pattern is similar to that of Taiwan, Hongkong, and Western countries.

## Background

The World Health Organization (WHO) encourages all countries to monitor the magnitude and causes of visual impairment in order to scrutinize and eliminate avoidable blindness [1]. Registered data can provide valuable information regarding blindness. Blindness registries mainly exist in developed countries and are held by state or partially state funded organizations that provide some kind of assistance to the visually disabled[2]. China is a developing country and cannot provide funds to establish a nationwide networked blindness registry. Also varied socioeconomic conditions affect the development of this project in different regions of China.

Shanghai is the largest metropolis in China and its GDP per capita reached $10,529 USD in 2008[3]. Jing-An district is among the nine downtown districts of Shanghai, where a public welfare system is most developed. Jing-An district established a blindness registry in 1992, which is held by the local Disabled Person's Federation (DPF), which is funded by the local government. Although registration is entirely voluntary, it confers significant practical and monetary benefits. We analyzed the data from the Jing-An district DPF with specific objectives including: (1) to identify the causes of registered blindness and to calculate the gender and age-specific prevalence of blindness; (2) to calculate the 3-year incidence of registered blindness in the most recent 9 years; and (3) to suggest priorities for research and intervention strategies for blindness.

## Methods

This study is based on the blindness data of the Jing-An district DPF. Age, gender and cause of visual disability data were collected and analyzed. The prevalence at the end of years 2003, 2007, 2009 and the 3-year incidence of blindness was calculated. The study was performed in accordance with the Declaration of Helsinki for research involving human subjects. The study met all standards for ethical approval in China, and the protocol was approved by the institutional board at the health bureau of Shanghai, China.

The criteria for registering blindness in China are in accordance with the WHO categories of visual impairment, and the category of interest for our study was that the visual impairment was irreversible [4]. Blindness was defined as a best spectacle corrected visual acuity (BSCVA) of less than 3/60 in the better eye, or a corresponding visual field loss to less than 10 degrees in the better eye with best possible correction.

All registered blindness cases need to undergo an assessment of visual impairment. The assessment system has been in place in Jing-An district central hospital from 1992 to the present. In the first 9 years, the causes of blindness were not recorded. From 2001, a comprehensive ocular examination was performed on every applicant including uncorrected visual acuity (UCVA), refraction, BSCVA, ophthalmoscope examination and retinal photography. Visual field tests were performed by a kinetic arctic perimeter (YZ22, 66 Vision Tech. Co., Ltd, China) when visual disability was due to glaucoma, retinitis pigmentosa (RP) and other optic nerve diseases.

The causes of blindness were classified according to the International Classification of Diseases, 10th edition [5]. Myopic macular degeneration (MMD) was considered only in subjects with a refractive error exceeding -6.0 diopters in either eye with one or more of the following ophthalmologic findings: tessellated fundus with yellowish white diffuse or grayish white patchy chorioretinal atrophy, macular hemorrhage or posterior staphyloma [6]. Age-related macular degeneration (AMD) was defined according to the Wisconsin age-related maculopathy grading system [7]. Early AMD was defined by the presence of either soft indistinct drusen or the presence of any type of drusen associated with retinal pigment epithelium depigmentation or increased retinal pigment. Late AMD was defined by the appearance of either exudative macular degeneration or pure geographic atrophy. Glaucoma was defined according to the International Society for Geographical and Epidemiological Ophthalmology classification [8]. Diagnosis of RP was based on night blindness, progressive loss of peripheral visual field, and decreased visual acuity with age, as well as on typical signs observed under fundus examination. The diagnosis of diabetic retinopathy, corneal opacity, and other diseases as causes of blindness followed the ophthalmology practice guidelines edited by the China Academy of Ophthalmology.

Using best judgement, the ophthalmologist attempted to identify the disorder causing the greatest limitation of vision as the cause of blindness. The causes of blindness in both eyes were recorded. When two causes appeared to have an equal contribution to visual impairment for one eye, the primary cause was assigned as the cause of blindness. If cataract was regarded as the main cause of blindness, the patient was referred for surgery and reassessed at least 2 months postoperatively if visual function restored unsatisfactorily.

Statistical analysis was performed using SPSS software version 13 (SPSS, Inc., Chicago, IL). The incidence of blindness caused by some diseases is low and varied in different years in a defined area, so we calculated the 3-year incidence of registered blindness per 100,000 in order to reduce the deviation of incidence in different year. And the prevalence of registered blindness per 100,000 were also calculated. Age was grouped as 1 y - 29 y, 30 y - 49 y, 50 y - 69 y and 70 y or older. Binary logistic regression was used to analyze the factors related to the occurrence of registered blindness. Odds ratios and 95% confidence intervals were determined to describe the influence of age and gender on the prevalence of visual impairment. A Chi-square test was used to analyze the difference between genders. P < 0.05 was considered as statistically significant.

## Results

### Prevalence and causes

The number of people in the blindness registry increased from 378 in 2003 to 458 in 2006 to 514 in 2009. The reported blindness also increased significantly from 114.7 per 100, 000 in 2003 to 145.8 per 100,000 in 2006 to 165.9 per 100,000 in 2009 (P < 0.05, P < 0.05, respectively). The age-specific prevalence and gender-specific prevalence for 2009 are summarized in Table 1. The association of sex and age with blindness was calculated with a logistic regression model, as shown in Table 2. Gender was not a significant factor, but age significantly affected the prevalence. The odd ratios (OR) were 2.57 in the 30 y - 49 y group (P < 0.001), 7.27 in the 50 y - 69 y group (P < 0.001), and 21.2 in the 70 y or older group (P < 0.001).

**Table 1:** Prevalence of blindness in Jing-An district, Shanghai, China in 2009

	Age(yrs)	population	blind	Prevalence	95%CI	
					lower	Upper
FEMALE						
	1-29	43152	15	34.8	17.2	52.3
	30-49	42425	22	51.9	30.2	73.5
	50-69	47173	109	231.1	187.7	274.4
	70 or 70+	25822	112	433.7	353.6	513.9
	Total	158572	258	162.7	142.9	182.5
MALE						
	1-29	44153	7	15.9	4.1	27.6
	30-49	40752	39	95.7	65.7	125.7
	50-69	47383	87	183.6	145.1	222.2
	70 or 70+	18893	123	651	536.3	765.7
	Total	151181	256	169.3	148.6	190.1
TT						
	1-29	87305	22	25.2	14.7	35.7
	30-49	83177	61	73.3	54.9	91.7
	50-69	94556	196	207.3	178.3	236.3
	70 or 70+	44715	235	525.6	458.5	592.6
	Total	309753	514	165.9	151.6	180.3

**Table 2:** Association of registered blindness with gender and age in Jing-an district in 2009

		OR	95%CI		P
			lower	Upper	
Sex	male	1			
	female	0.87	0.73	1.04	0.12
age	1-29	1			
	30-49	2.57	2.13	3.11	<0.001
	50-69	7.27	5.48	9.63	<0.001
	70 or 70+	21.20	13.69	32.84	<0.001

CI: confidence interval

OR: odds ratio (binary logistic regression)

The leading causes of blindness that were registered in 2001-2009 are summarized in Table 3. The causes of blindness were MMD (19.4%), followed by glaucoma (17.7%), AMD (11.8%), optical nerve atrophy (9.4%), RP (8.6%), diabetic retinopathy (7.8%) and corneal opacity (5.8%).

**Table 3:** Causes of new registered blindness in Jing-An district, Shanghai, China in 2001-2009

	2001-2003					2004-2006					2007-2009				
Causes	cases	eyes	%	order	Incidence 1/100000	cases	eyes	%	order	Incidence 1/100000	case	eyes	%	order	Incidence 1/100000
MMD	15	29	14.5	2	4.8(2.4,7.3)	17	32	15.1	1	5.5(2.9,8.1)	39	74	26.1	1	12.6(8.6,16.5)
Glaucoma	19	38	19.0	1	6.1(3.4,8.9)	16	32	15.1	1	5.2(2.6,7.7)	28	53	18.7	2	9.0(5.7,12.4)
AMD	13	20	10.0	5	4.2(1.9,6.5)	16	29	13.7	3	5.2(2.6,7.7)	19	33	11.6	3	6.1(3.4,8.9)
RP	6	11	5.5	7	1.9(0.4,3.5)	13	26	12.3	4	4.2(1.9,6.5)	12	23	8.1	4	3.9(1.7,6.1)
Diabetic	8	16	8.0	6	2.6(0.8,4.4)	8	16	7.6	6	2.6(0.8,4.4)	11	22	7.8	5	3.6(1.5,5.6)
CO	3	4	2.0	9	1.0(0,2.1)	10	15	7.1	7	3.2(1.2,5.2)	16	21	7.4	6	5.2(2.6,7.7)
RD	15	27	13.5	3	4.8(2.4,7.3)	5	8	3.8	8	1.6(0.2,3.0)	10	16	5.6	7	3.2(1.2,5.2)
ONA	13	26	13.0	4	4.2(1.9,6.6)	14	25	11.8	5	4.5(2.2,6.9)	7	14	4.9	8	2.3(0.6,3.9)
UVITIS	3	6	3.0	8	1.0(0,2.1)	4	7	3.3	9	1.3(0,2.6)	6	10	3.5	9	1.9(0.4,3.5)
OTHERS	17	23	11.5		5.5(2.9.8.1)	12	22	10.4		3.9(1.7,6.1)	16	18	6.3		5.2(2.6,7.7)
Total	100	200	100.0		32.3(25.9,38.6)	106	212	100.0		34.2(27.7,40.7)	142	284	100.0		45.8(38.3,53.4)

MMD: myopic macular degeneration; AMD: age-related macular degeneration; ONA: optic nerve atrophy; RP: retinitis pigmentosa; CO: corneal opacity; RD: retinal detachment.

### 3-year incidence

The 3-year incidence of registered blindness increased from 32.3 (range 26.0-38.6) per 100,000 in 2001-2003 to 34.2 (range 20.7-40.7) per 100,000 in 2004-2006 to 40.8 (range 40.1-55.5) per 100,000 in 2007-2009 (Table 4). In the blindness registry, retinal detachment was the third cause in 2001-2003, but became the eighth in 2004-2006 and the seventh cause in 2007-2009. Optic nerve atrophy was the fourth cause of blindness in 2001-2003, and became the seventh cause in 2004-2006 and the eighth cause in 2007-2009. However, AMD was the fifth cause in 2001-2003, the third in 2004-2006, and the third in 2007-2009.

**Table 4:** Prevalence and 3-years-incidences of blindness in Jing-An district, Shanghai, China.

	2001-2003	2004-2006	2007-2009
Population(10,000)	33.24	30.96	30.97
Blind	378	458	514
Prevalence rate (95%CI)(1/100,1000)	113.7(102.3-125.2)	147.9(134.4-161.5)	165.9(151.6-180.3)
New blind	100	106	142
3-year-incidence (95%CI) (1/100,1000)	32.3(26.0-38.6)	34.22(27.7-40.7)	47.78(40.1-55.5)

### Age and new blindness

The ages of the registered cases of new blindness is summarized in Table 5. The average registered age was 64.19 ± 15.86 y. The average registered age increased from 60.1 ± 17.3 y in 2001-2003 to 65.4 ± 15.2 y in 2004-2006 to 66.1 ± 14.8 y in 2007-2009 (P < 0.05, P < 0.05, respectively). The main reason for the increased age of new blindness was that there was an increase in the age of the registered blind due to MMD, optic nerve atrophy, corneal opacity and AMD.

**Table 5:** Registered age of new blindness in Jing-An district, Shanghai, China.

	2001-2009	2001-2003	2004-2006	2007-2009
	Mean ± 1SD	Mean ± 1SD	Mean ± 1SD	Mean ± 1SD
Glaucoma	67.30 ± 14.61	65.89 ± 14.01	67.94 ± 16.23	67.89 ± 14.52
Diabetic	63.00 ± 13.79	63.38 ± 13.73	58.00 ± 14.14	63.64 ± 14.92
MMD	61.32 ± 12.22	54.60 ± 16.61	61.12 ± 11.36	64.00 ± 9.68*
RP	57.29 ± 13.83	58.00 ± 13.28	59.15 ± 10.16	54.92 ± 17.83
AMD	75.31 ± 9.42	71.00 ± 8.98	77.70 ± 9.28*	75.74 ± 9.31
ONA	59.25 ± 17.43	53.23 ± 19.74	66.69 ± 12.24*	53.43 ± 18.82
CO	67.52 ± 16.55	43.00 ± 33.87	66.40 ± 11.50*	72.81 ± 11.33*
RD	61.97 ± 13.78	57.47 ± 15.58	68.80 ± 8.81	65.30 ± 11.28
UVITIS	62.77 ± 14.93	60.33 ± 14.15	59.25 ± 11.95	66.33 ± 18.45
OTHERS	59.93 ± 23.18	58.44 ± 21.51	54.18 ± 27.03	65.73 ± 22.16
Total	64.19 ± 15.86	60.10 ± 17.29	65.43 ± 15.24*	66.12 ± 14.82*

MMD: myopic macular degeneration; AMD: age-related macular degeneration ONA: optic nerve atrophy; RP: retinitis pigmentosa; CO: corneal opacity; RD: retinal detachment.

*:P < 0.05 compared to in 2001-2003

## Discussion

To our knowledge, this is the first report on registered blindness in China. Because of the varied socioeconomic levels in China, and the independence of registries in different regions, it is impossible to obtain a complete national dataset, as well as a complete dataset for Shanghai. We therefore analyzed limited data based on the blindness registry in Jing-An district, Shanghai, China.

The number of people in the blindness registry in Jing-An district has increased by 36% since 2003 and by 13% since 2007. In contrast, the population of the district remained stable. The increase in the prevalence and incidence of blindness during 2001-2009 likely represents increased registration rather than increasing levels of disease, considering the short time period involved. Higher life expectancy in the general population in Shanghai may have contributed as well; according to an official publication of the Shanghai Bureau of Statistics, life expectancy in 2001 was 79.7 y and 81.3 y in 2009[3].

Currently, the overall prevalence of blindness is 0.17% for the total population, 0.21% for ages 50 y - 69 y, and 0.53% for ages 70 y or older. The prevalence in this study is lower than that reported in most studies based on a sample of the population in urban China (Table 6) [6,9-19]. This is possibly due to inclusion of blindness due to cataracts in the studies. A recent study showed the prevalence of blindness was 0.61% for ages 70 y or older in Shanghai and 29.4% was due to cataract [17], which is a prevalence rate closer to our results. Another study in Henan province showed that the prevalence of blindness in urban areas was only 0.09% [18]. To our knowledge, there are no data available on the incidence of blindness in China to compare with this study. It is difficult to compare the incidence and prevalence of blindness with other countries because of different definitions of blindness and difference age distributions. The prevalence of registered blindness was 0.17% - 0.22% in Ireland (blindness was defined as BSCVA in the better eye of less than 6/60)^2 ^and 0.26% - 0.32% in Israel (using WHO criteria) [20]. The incidence of registered blindness was 0.036% - 0.029% in Israel (using WHO criteria), 0.02% in Ireland (using US criteria) and 0.012% in southern Germany (blindness was defined as BSCVA in the better eye of less than 1/50) [20-22]. In our study, blindness was defined by WHO criteria and cataract was extirpated as the cause of registered blindness. Young babies were also enrolled in this study, which would decrease the prevalence and incidence of blindness.

**Table 6:** Prevalence of blindness and its causes reported from population based studies in china

Country	Age(yrs)	Prevalence	Main causes
Beijing, China (Xu L et al.2006)	> = 40	0.3%	Cataract(38.5%); Cornea opacity (15.4%);Myopic macular degeneration(7.7%); Glaucoma(7.7%)
Handan china (Liang YB et al. 2008)	> = 30	0.5%	Cataract (36.6%);Myopic macular degeneration (19.5%) Glaucoma(7.3%);Cornea opacity(7.3%)
Taiwan, China (Hsu WM, et al. 2004)	> = 65	0.59%	Cataract (41.7%);Myopic macular degeneration (12.5%) AMD(10.4%)
Hongkong, China (Michon JJ, et al. 2002)	> = 60	1.8%	Cataract (51.7%);Macular degeneration (27.1%) Glaucoma(7.1%)
Hebei, China (Song XJ.1992)		0.19% in urban areas	Cataract(32.3%);trachoma complications(13.4%) corneal disease(13.06%);glaucoma(9.97%)
Shanxi, China (Zhang JX.1990)		0.18% in cities	Cataract (29.7%)
Shangdong, china (Yu XM.1992)		0.34%	Cataract, corneal diseases and glaucoma in urban areas
China (Zhang SY.1992)		0.43%	Cataract(41.06%);Corneal disease(15,38%) Trachoma (10.4%);Glaucoma (8.8%)
Chongqing, china (Liu S, et al. 2007)	>50	1.8%	Cataract (17.9%);Retinal diseases (20.7%); refraction error(15.4%); cornea disease (11.6%); glaucoma (13.4%)
Shanghai (Huang XB, et al. 2009)		0.95% in 60-79 yrs 0.68% in 70-79 yrs 0.40%yrs in 80 yrs or older	Macular degeneration(28.57%);Cataract(27.27%); Corneal opacity (6.49%); Retinal detachment (5.84%); Optical nerve atrophy (5.19%); Retinitis pigmentosa (5.19%)
Henan (Li Y et al.2009)		0.09% in urban	no noted
Hubei (Chen W et al. 2009)		0.33% in urban	Cataract (54.55%);Glaucoma(10.22%); Congenital abnormal (8.41%);Corneal disease(7.48%); Retinal and choroidal disease (6.54%)

The leading causes of blindness in the present study were MMD (22.6%), glaucoma (18.7%), AMD (11.6%) and RP (8.1%). Some studies showed that corneal opacity was also a major cause of blindness in China (shown in Table 5) [9,12-18]. In the present study corneal opacity was the eighth cause of blindness. Although the number of cases of corneal opacity increased, along with an increase in the registered age from 43.0 ± 33.9 y in 2001-2003 to 72.8 ± 11.3 y in 2007-2009, visual acuity must have been lost in an earlier stage. The reason for this is that infectious keratitis and trachoma have been well controlled in Shanghai. A recent study showed that glaucoma was the eighth cause of blindness and accounted for 4.1% of blindness in Shanghai, whereas it was the second cause at 18.7% in the present study [17]. However, no visual field examinations were performed in their study. AMD has increased dramatically as a cause of blindness. It was the fifth cause in 2001-2003, while it became the third cause in 2007-2008. The pattern of causes of blindness was similar to that in Taiwan, Hongkong, and Western countries [6,11,20-22]. Glaucoma and AMD have also become the leading causes of blindness in the Jing-An district of Shanghai.

The analysis shows the importance of glaucoma as the leading cause of blindness in China today. Most patients with glaucoma can maintain sufficient visual acuity if effective treatment is given at an early stage, especially for primary angle-closure glaucoma (PACG), which is major category of glaucoma in China. The study showed that 92.86% of blindness due to glaucoma occurred at 51 y or older. We therefore suggest that local health bureaus should conduct glaucoma screening for those aged 51 y or older to diagnose glaucoma at an early stage.

AMD and MMD are the chief causes leading to blindness. Unfortunately, for most people with AMD, vision loss can neither be prevented nor adequately reversed. There is clearly a need for further research on the causes and risk factors of AMD. Persons with MMD will also benefit from the research. Controlling the development of high myopia is impending. Research on the mechanisms of myopia progression is necessary to decrease the vision impairment burden of students.

This study is based on registered data for a defined geographic area. We acknowledge there are some limitations in our study. We had to rely on the limited information from the CDPF, and the causes of blindness registered before 2001 were not recorded. The increase in the prevalence and incidence of blindness likely represents increased registration rather than increasing levels of disease. In this study, the cause of blindness was recorded for each eye, which would increase the proportion of disorders with a bilateral tendency such as AMD, MMD and glaucoma as the cause of blindness. It would be better to record the causes by case. The efficacy of the registration system is not certain. Previous studies in other countries found that only 45% - 60% of eligible patients were registered [23]. It is therefore necessary to make a dedicated effort to evaluate the efficacy of the registry system in China.

## Conclusions

The 3-year incidence and reported blind increased in the past 9 years. The leading causes of new cases of blindness that were registered were MMD, glaucoma and AMD. The pattern of causes has changed little in the past 9 years, is different from other locations in China, and is similar to patterns in Taiwan, Hongkong and Western countries.

## Abbreviations

WHO: The World Health Organization; DPF: Disable Person's Federation; BSCVA: best spectacle corrected visual acuity; UCVA: uncorrected visual acuity; MMD: Myopic macular degeneration; AMD: Age-related macular degeneration; RP: Diagnosis of retinitis pigmentosa.

## Competing interests

The authors declare that they have no competing interests.

## Authors' contributions

LW carried out the data collection, performed data analysis and drafted the manuscript. XS participated in the design of the study. XZ and CW participated in the study design and coordination and helped to draft the manuscript. All authors read and approved the final manuscript.

## Pre-publication history

The pre-publication history for this paper can be accessed here:

http://www.biomedcentral.com/1471-2415/11/10/prepub
